# Discovery of novel DNA methylation biomarkers for non‐invasive sporadic breast cancer detection in the Latino population

**DOI:** 10.1002/1878-0261.12842

**Published:** 2020-11-19

**Authors:** Mónica Cappetta, Lucía Fernandez, Lucía Brignoni, Nora Artagaveytia, Carolina Bonilla, Miguel López, Manel Esteller, Bernardo Bertoni, María Berdasco

**Affiliations:** ^1^ Departamento de Genética Facultad de Medicina Universidad de la República Montevideo Uruguay; ^2^ Departamento Básico de Medicina Facultad de Medicina Universidad de la República Montevideo Uruguay; ^3^ Departamento de Medicina Preventiva Facultad de Medicina Universidad de São Paulo Brazil; ^4^ Population Health Sciences Bristol Medical School University of Bristol UK; ^5^ Cancer Epigenetics and Biology Program (PEBC) Bellvitge Biomedical Research Institute (IDIBELL) Barcelona Spain; ^6^ Epigenetic Therapies Group, Experimental and Clinical Hematology Program (PHEC) Josep Carreras Leukaemia Research Institute (IJC) Badalona Spain; ^7^ Cancer Epigenetics Group, Cancer and Leukemia Epigenetics and Biology Program (PEBCL) Josep Carreras Leukaemia Research Institute (IJC) Badalona Spain; ^8^ Physiological Sciences Department School of Medicine and Health Sciences University of Barcelona Spain; ^9^ Centro de Investigación Biomédica en Red Cáncer (CIBERONC) Madrid Spain; ^10^ Institució Catalana de Recerca i Estudis Avançats (ICREA) Barcelona Spain

**Keywords:** biomarker, breast cancer, DNA methylation, human diversity, non‐invasive test

## Abstract

Human diversity is one of the main pitfalls in the development of robust worldwide biomarkers in oncology. Epigenetic variability across human populations is associated with different genetic backgrounds, as well as variable lifestyles and environmental exposures, each of which should be investigated. To identify potential non‐invasive biomarkers of sporadic breast cancer in the Uruguayan population, we studied genome‐wide DNA methylation using Illumina methylation arrays in leukocytes of 22 women with sporadic breast cancer and 10 healthy women in a case–control study. We described a panel of 38 differentially methylated CpG positions that was able to cluster breast cancer patients (BCP) and controls, and that also recapitulated methylation differences in 12 primary breast tumors and their matched normal breast tissue. Moving forward, we simplified the detection method to improve its applicability in a clinical setting and used an independent well‐characterized cohort of 80 leukocyte DNA samples from BCP and 80 healthy controls to validate methylation results at specific cancer‐related genes. Our investigations identified methylation at *CYFIP1* as a novel epigenetic biomarker candidate for sporadic breast cancer in the Uruguayan population. These results provide a proof‐of‐concept for the design of larger studies aimed at validating biomarker panels for the Latin American population.

## Graphical abstract

Epigenetic alterations are already being incorporated as robust biomarkers for prediction of cancer risk. Despite this success, validation in specific populations with different genetic backgrounds and variable lifestyle and environmental exposures is necessary. In this paper, we investigate the potential of blood DNA methylation as a non‐invasive test for detection of sporadic breast cancer biomarker in the Latin American population.

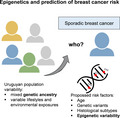

**Abbreviations**
BMI: body mass index
CpGDMs: differentially methylated CpG sites
MS‐HRM: methylation‐sensitive high‐resolution melting

## 1 Introduction

Breast cancer is a complex and heterogeneous disease caused by the interactions of both genetic and non‐genetic factors. Age, gender, and family history are the major factors for breast cancer. The known high‐risk inherited mutations in breast cancer susceptibility genes, such as *BRCA1*, *BRCA2*, *P53*, *PTEN*, *CHEK2*, and *ATM*, together only explain 1.5–3% of all breast cancer cases. Meanwhile, known variants with low‐penetrance risk to breast cancer only represent a predictive accuracy of 60% [1]. Therefore, genetic risk factors are not enough to evaluate risk of breast cancer.

DNA methylation is a key process involved in the regulation of gene expression. Interestingly, DNA methylation is potentially modifiable and is related to age, the strongest breast cancer risk predictor [2]. Alterations in DNA methylation patterns, both at the global genomic level and loci‐specific, have been successfully explored as molecular biomarkers in cancer management [3]. In our previous work, we reported global DNA hypomethylation in leukocytes of sporadic breast cancer patients (BCP) compared with healthy controls, supporting the potential use of DNA methylation in leukocytes as a biomarker for cancer [4]. Additionally, we found a negative correlation between African ancestry and global DNA methylation in cancer patients, suggesting that the ancestral genome structure generated by the admixture process in the Uruguayan population influences DNA methylation patterns [4]. This underscores the importance of searching for population‐specific DNA methylation markers for sporadic breast cancer.

In addition, most studies that leverage DNA methylation as potential biomarkers for cancer use primary tumor tissues. However, a reliable biomarker should meet criteria such as acceptable costs and feasibility in preventive medicine to stratified population according to risk scores or to detect cancer earlier. Detection of CpG methylation changes in non‐ or minimal invasive liquid biopsies including blood cells, saliva, or urine would increase translation of molecular evidence into clinical practice [3]. Several studies have investigated peripheral blood DNA methylation biomarkers in different cancer types including head and neck, breast, lung, bladder, gastric cancer, prostate, colorectal, and ovarian cancers [5, 6, 7, 8, 9, 10]. Few studies have attempted to investigate the role of loci‐specific DNA methylation in leukocytes as a marker of breast cancer, most of them by candidate gene approaches, and did not use a validation set to confirm their results (reviewed in Ref. [11, 12]). However, the majority of the genome‐wide studies were carried out in Europe, Asia, and USA and must be validated in each population, especially in those with admixed genetic ancestry like the Latino population [13].

In the current study, we aimed to identify novel candidate sporadic breast cancer epigenetic biomarkers in peripheral blood from the Uruguayan population. Consequently, we analyzed genome‐wide DNA methylation signatures in leukocytes from sporadic BCP and healthy women in a well‐defined discovery cohort in a case–control study. Epigenetic biomarkers observed in specific candidate genes were replicated using a large validation cohort.

## 2 Materials and methods

### 2.1 Study population

For genome‐wide DNA methylation profiling analysis, 24 DNA samples from peripheral blood leukocytes of patients with sporadic breast cancer and 12 DNA samples from unaffected controls were selected from a group of individuals originally recruited in a previous study described in Bonilla *et al*. [14]. After a quality control analysis, a discovery cohort composed of 22 patients and 10 controls were included in the methylation study (Table S1). To validate candidate CpG sites, we selected a large and independent validation cohort of the same previous study consisting of 80 DNA leukocyte samples from sporadic BCP and 80 DNA leukocyte samples from healthy controls, paired by age, socioeconomic status, and educational level (Table S2). The procedures followed were approved by the ethics committee of the Facultad de Medicina of the Universidad de la República, Uruguay (Reference number: 071140‐000303‐12). After obtaining written informed consent from all participants of the study, peripheral blood was drawn for DNA extraction and participants answered an interview‐based questionnaire to record medical and epidemiological information. All human samples included in the study were handled in accordance with the tenets of the Declaration of Helsinki.

All cases were originally selected according to the following criteria: women with breast cancer over 35 years with no personal history of cancer and no first‐degree family history of breast and/or ovarian cancer, and non‐consanguineous parents. All patients were sampled at the time of diagnosis and prior to surgery and/or any therapy. The control group was women over 35 years of age, with normal mammograms, no first‐ or second‐degree family history of cancer, and unrelated to any other project participant. Controls were selected in the same hospitals as patients. For inclusion in this study, all participants were required to: (a) have a normal hemogram at the time of sampling, and (b) have previous data of global DNA methylation quantification by HPLC and individual genetic ancestry [4]. Breast cancer cases and controls were age‐ and individual genetic ancestry‐paired.

Genetic ancestry of all participants of this study was previous determined by 59 ancestry informative markers (AIMs) selected from the AIMs panel for Hispanic population described by Fejerman *et al*. [15]. Genotyping and individual admixture analysis were described in Cappetta *et al*. [4].

Of all the epidemiological information collected from the participants of the discovery and validation cohorts, the following variables were analyzed in this study: age, sex, genetic ancestry, body mass index (BMI), smoking status (self‐reported), and tumor characteristics (Table S1,S2).

### 2.2 Genome‐wide methylation analysis

Infinium HumanMethylation450 BeadChips were used to analyze DNA methylation on a genome‐wide scale in the discovery cohort. DNA was extracted from whole peripheral blood by standardized salting out methods. Five hundred nanogram of DNA per sample was first bisulfite treated using the Zymo EZ‐96 DNA‐methylation kit (Zymo Research, Orange, CA, USA). Next, about 200 ng of bisulfite‐converted DNA was used for hybridization on the HumanMethylation450 BeadChip (Illumina, San Diego, CA, USA) according to the manufacturer's protocol. Pre‐processing and initial quality assessment of hybridizations was performed using the Illumina BeadStudio methylation module software, version 3.2 (Illumina, Inc.). Negative control bead types were used to obtain an estimation of the background intensity level that was subtracted from the loci probe signals. Moreover, the distribution of intensities at negative controls was used to compute detection *P*‐values which were assigned to each probe as a measure of the signal‐to‐noise ratio. As quality criteria, probes showing a detection *P*‐value greater than 0.001 in at least 6% of the samples and samples with a detection *P*‐value greater than 0.001 in more than 10% of the probes were considered defective. Thousand nine hundred and forty‐seven probes (CpG sites evaluated) did not reach these criteria and were therefore excluded from further analysis. Beta values were then computed from methylated and unmethylated signals from each microarray assay, and potential bias of dye in the array was corrected using the methylumi R package [16] from Bioconductor [17]. The methylation percentage of a CpG site was reported as a beta value ranging between 0 (no methylation) and 1 (full methylation). Once the normalized beta values were obtained, a second phase of quality control was performed. *Β* values with a *P*‐value detection > 0.01 and 23631 CpG sites exhibiting SNP with a frequency of > 1% (1000 Genomes Project Consortium 2010) in the probe sequence were removed from subsequent analyzes. Since all DNA samples were from women, the X chromosome probes were not removed. As a result of the quality control process, we included in subsequent analysis 459 999 probes (CpG sites) in 22 women with breast cancer and 10 control women. The Infinium methylation data are available in the Gene Expression Omnibus (GEO) database under the accession number GSE148663.

### 2.3 Independent public cohorts for *in silico* studies

Methylation data for the independent European BCP were obtained from the public data set GSE52865 [18]. This data set provided methylation from breast tumor tissue samples and normal breast tissue of European women hybridized to the Illumina HumanMethylation450 BeadChip array. We analyzed only 24 samples corresponding to breast tumor tissue samples and their paired adjacent normal breast tissue from this data set. Clinical characteristics of these samples were provided in Table S3.

DNA methylation and clinical data of 735 BCP of The Cancer Genome Atlas (TCGA) database were abstracted from GDC Data Portal of the National Cancer Institute (https://portal.gdc.cancer.gov/). We analyzed methylation data of 735 breast primary tumors and 89 normal breast tissues.

### 2.4 Statistical analysis

All statistical analyses were carried out using the r programming language (http://www.r‐project.org/) and no parametrical analyses. To identify consistently differentially methylated CpG sites (CpGDMs) between BCP and controls, Wilcoxon rank sum test was performed for normalized beta values of each group. The *P*‐values were adjusted for multiple testing using false discovery rate estimation (FDR), and those CpGs with *P*‐value < 0.05 were selected and termed ‘CpGDMs’.

Cluster analysis of the selected CpGDMs was performed using unsupervised hierarchical clustering with complete linkage and Euclidean distance as a measure of similarity between samples. We then used the pvclust package in R using multiscale bootstrap resampling (*n* = 10 000) to define statistically significant samples clusters [19]. Furthermore, in order to explore the similarity of methylation data between BCP and unaffected control groups, an analysis of multidimensional scaling was performed using variable methylation values (β value) of CpGDMs of each individual.

Possible confounding effects on methylation status were evaluated using generalized linear regression models including age, genetic ancestry, smoking status, and leukocyte cell composition as covariables. The *estimateCellCounts* function of the *minfi* Bioconductor package was utilized to determine the proportions of the white blood cell types (CD4+ and CD8+ T cells, CD56+ NK cells, B cells, monocytes, and granulocytes) in each sample [20].

Age, BMI, and genetic ancestry were analyzed as a continuous variable and were assessed using Student's *t*‐test, while the remaining parameters of the study were considered qualitative variables. Smoking status was categorized as yes/no considering the individual as a smoker whether it was at the time of sampling and/or in the past. Data obtention was not possible for all individuals included in the study.

Association between clusters in BCP and epidemiological or clinical covariates (tumor stage, tumor histological type, hormone receptors) was assessed using Kruskal–Wallis test and Fisher's exact test.

Overall survival for BCP from the TCGA database was evaluated using Kaplan–Meier analysis grouping patients with methylation values above and below the median, and a proportional hazard Cox regression model adjusted for the methylation level of the candidate biomarker evaluated and age at diagnosis of the disease.

The receiver operating characteristic (ROC) curve was plotted with R package pROC version 1.16.1 [21], to estimate the discriminatory power of methylation at the candidate region of the *CYFIP1* gene. The area under the ROC curve (AUC) was calculated, and the DeLong method was used to calculate the 95% confident interval (CI) for AUC.

### 2.5 Enrichment analyses of biological pathways and common sequence features

The genes associated with CpGDMs sites were mapped to discern their relation to cancer by gene ontology analysis using Genes to Systems Breast Cancer Database (G2SBC, http://www.itb.cnr.it/breastcancer/) [22] and searching in the COSMIC database (https://cancer.sanger.ac.uk/cosmic/) [23]. The Database for Annotation, Visualization, and Integrated Discovery (DAVID v6.8) [24] and Kyoto Encyclopedia of Genes and Genomes (KEGG database) were used for an analysis of molecular pathways. In the DAVID analysis, the set of genes represented on the Illumina HumanMethylation450 array was used as the reference set and the set of CpGDMs composed the gene set tested. Using the manifest file provided by Illumina, we classified CpGDMs according to their position relative to CpGs islands (Island, Shore, Shelf or Open sea) or relative to repetitive elements; or their genomic compartments feature (Promoter, TSS, Exon, intron, intergenic region). The genomic location of the CpGDMs was compared to the distribution of the CpGs in the whole methylation data set. *P* values were computed using Fisher's exact test to determine over‐ or under‐representation of the CpGDMs.

### 2.6 Bisulfite‐treated DNA sequencing

We amplified by PCR four CpGDMs using bisulfite‐treated DNA from four BCP and four controls. PCR amplification reactions in a final volume of 15 µL, containing: 10× PCR buffer EcoStart (Ecogen), 50 mm MgCl_2_ (Ecogen, Barcelona, Spain), 2 mm dNTPs; 1 µm specific primers to amplify the gene sequence and 3 U of DNA polymerase enzyme (DNA polymerase EcoStart; Ecogen). Primers were designed to amplify the selected CpGDM region and flanking sequences of the transcription start site of the corresponding gene (Table S4). PCR amplified sequences were visualized by electrophoresis and extracted from the agarose gel using the QIAquick Gel Extraction Kit (Qiagen, Hilden, Germany). The extracted DNA was cloned into competent *Escherichia coli* bacteria (NovaBlue SinglesTM) using the pGEM‐T® vector. Estimation of methylation status of each CpG site was performed by automated sequencing of 10 colonies of each study sequence (Applied Biosystems, Waltham, MA, USA). The average methylation throughout the region assessed for each gene between patients and controls was compared using Student's *t*‐test.

### 2.7 Methylation‐sensitive high‐resolution melting (MS‐HRM) assay

DNA obtained from blood samples was subjected to bisulfite modification using the EZ DNA methylation gold kit (Zymo Research) following the manufacturer's protocol. Primers used for amplifying flanking regions of cg14024502, cg26568226, cg04890607, cg09580608, cg01229567, cg19246761 and cg24840062 are listed in Table S4. PCR amplification and MS‐HRM assay were performed on Eco Illumina real‐time PCR. The final volume of each reaction system was 10 µL, including 5 µL of 2× Epitec HRM PCR Master Mix (Qiagen), 10 ngr of sodium bisulfite‐modified template DNA, and 0.4 mm of each forward and reverse primer. A series of methylated DNA standards (100%, 75%, 50%, 25%, 15%, 10%, 5%, 2.5% and 0% methylated DNA) were constructed by mixing universal unmethylated (0% methylated) and methylated (100% methylated) human whole genomic DNA samples (Qiagen). Fluorescence of each sample was normalized as a differential signal against unmethylated DNA control. Area under the curve from the normalized, difference curves was used to generate a standard curve and determine the degree of methylation of each DNA sample [25, 26].

To identify differential methylation in flanking regions of candidate CpGDMs between patients with breast cancer and controls in the validation cohort, Wilcoxon rank sum test was performed for methylation data from MS‐HRM analysis of each group. Possible confounding effects on methylation status in candidate genes were evaluated using generalized linear regression models including age, BMI, smoking status, and genetic ancestry.

## 3 Results

### 3.1 Blood DNA methylation profiling reveals a panel of differentially methylated CpGs that discriminates sporadic breast cancer patients and healthy controls

We applied a case–control study to describe the key genomic sequences involved epigenetically in the susceptibility to sporadic breast cancer in the Uruguayan population. Consequently, we performed genome‐wide DNA methylation profiling in a discovery cohort using DNA from leukocytes of women with sporadic breast cancer (*n* = 22) and healthy women as control (*n* = 10). Comparison of mean methylation values of all CpGs sites analyzed between BCP and control groups showed a high correlation across all CpGs (Spearman, *r* ^2^ = 0.997, *P* < 2.2 × 10^−16^), indicating that global DNA methylation patterns in all samples are very similar (Fig. S1A). In addition, to avoid spurious relationships due to technical or sampling procedures we applied hierarchical clustering of samples using the methylation values of random 45 000 CpG sites and we were unable to cluster samples according to their disease status (Fig. S1B).

**Fig. 1:**
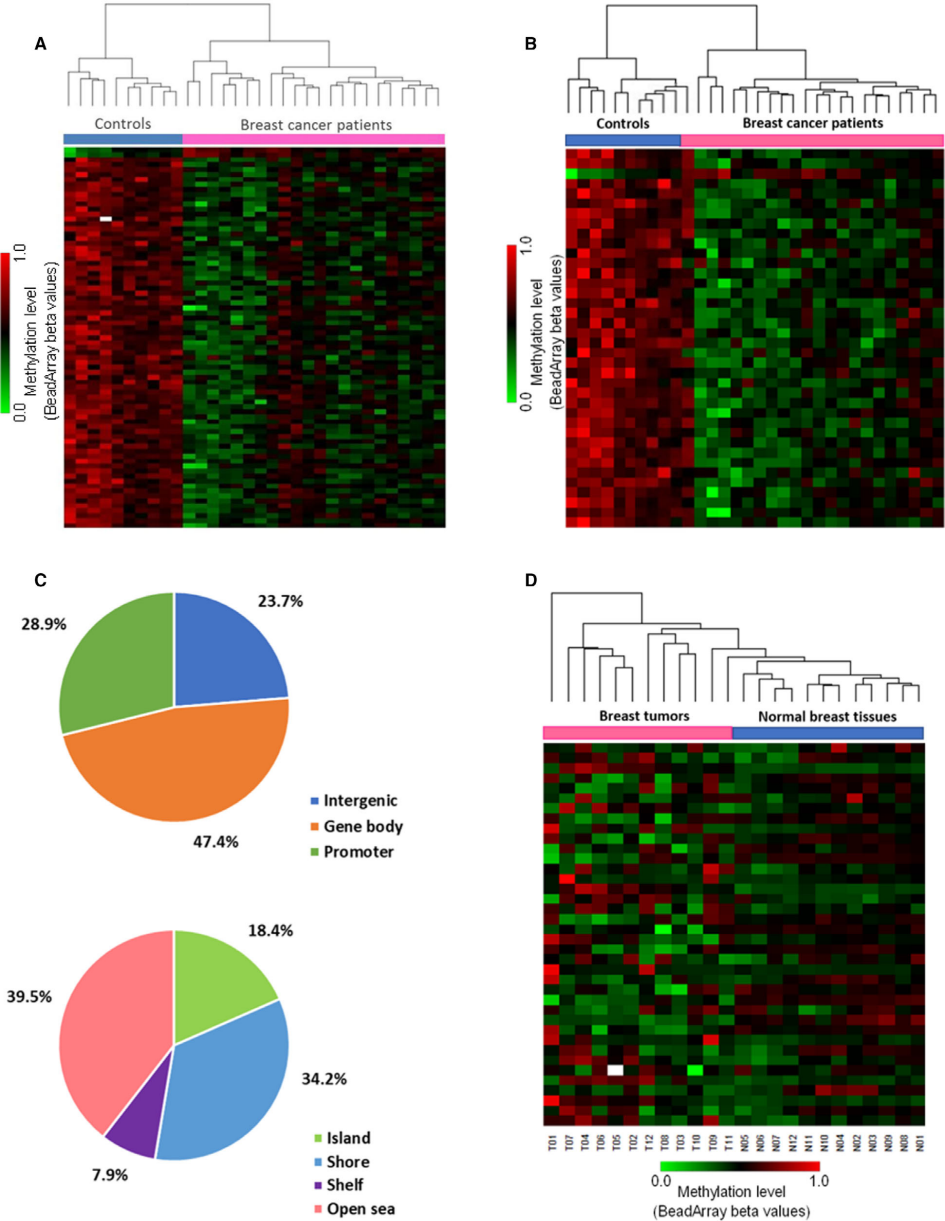
Definition of genome‐wide CpG profiles in sporadic BCP. (A) Hierarchical heatmap clustering of 77 CpGDMs in 22 BCP (purple) and 10 healthy controls (blue) analyzed on methylation array. CpGDM were ordered by the difference in mean betas values between patients and controls. Methylation level is color coded (green: lowest methylation level; red: highest methylation level). (B) Hierarchical heatmap clustering of 38 selected candidate CpGDMs in 22 BCP (purple) and 10 healthy women (blue). Candidate CpGDMs were ordered by the difference in mean betas values between patients and controls. Methylation level is color coded (green: lowest methylation level; red: highest methylation level). (C) Genomic distribution of 38 candidate CpGDMs regarding their respective location to genes and CpG context. (D) Hierarchical heatmap clustering of 38 candidate CpGDMs in 12 primary breast tumor samples (T, purple) and their matched normal breast tissues (N, blue) analyzed on methylation array. Methylation level is color coded (green: lowest methylation level; red: highest methylation level).

To identify CpGDMs between BCP and healthy controls, we applied a Wilcoxon rank sum test, determining 77 CpGDMs positions after correction for multiple testing (Table 1). This panel of identified CpGDMs was able to cluster BCP and healthy controls separately using a hierarchical cluster approach (Fig. 1A). These results indicate the existence of CpG methylation differences at specific sequences between cancer patients and controls at the leukocyte level, which can be easily visualized by unsupervised classification techniques and could function as a breast cancer signature in blood. Since we detected 3 defined subclusters among the patient samples group (Fig. 1A), we analyzed whether these subclusters were associated with tumor characteristics or epidemiological variables. However, no association was found in samples from cancer patients between age (*P* = 0.868), smoking status (*P* = 0.852), genetic ancestry (European *P* = 0.064; African *P* = 0.675; Native American *P* = 0.898), tumor stage (*P* = 0.716), histological type of tumor (*P* = 0.187), hormone receptors (ER *P* = 1; PR *P* = 0.112; Her2 *P* = 0.494) and the subgroups derived from cluster analysis.

**Table 1:** Features of 77 differentially methylated CpG (CpGDM) among BCP and unaffected control group. CpG: identification of the probe (from array). CHR: chromosome. TSS1500: 1500 bp of the start site of transcription. TSS200: 200 bp of the start site of transcription.

CpG	CHR	UCSC REFGENE	Genomic context	CpG island context	Δ β value (cancer patients ‐ controls)	*P* value (FDR)	*P* value adjusted*
cg01015663	1	*TCEA3*	gene body	Open sea	−0.0766	0.0494	**0.0123**
cg01229567	1	*MIB2*	TSS1500	Shore	−0.1184	0.0494	**0.0417**
cg04400047	1	*UBIAD1*	TSS1500	Shore	−0.0526	0.0494	**0.0343**
cg06432479	1	*TAL1*	gene body	Island	−0.0657	0.0494	**0.0300**
cg10159215	1	*LPPR5*	TSS200	Island	−0.1014	0.0498	0.1404
cg14024502	1	*MAP3K6*	TSS1500	Shore	−0.008	0.0498	**0.0275**
cg15452381	1	*HIVEP3*	5'UTR	Open sea	−0.0196	0.0498	**0.0095**
cg16727538	1	*C1orf213*		Shore	−0.0714	0.0494	**0.0271**
cg19246761	1	*MIB2*	TSS1500	Shore	−0.1116	0.0494	**0.0353**
cg26251270	1	*GPX7*	TSS1500	Shore	−0.0571	0.0494	**0.0197**
cg26750487	1	*CR1L*	TSS200	Island	−0.0848	0.0494	0.9985
cg02537909	2	*COBLL1*	gene body	Island	−0.0604	0.0494	0.9979
cg02738156	2	*LINC00487*	intergenic	Open sea	−0.0785	0.0494	0.0807
cg09408768	2	*KCNJ3*	TSS200	Island	−0.0621	0.0494	0.0635
cg17165836	2	*AFF3*	gene body	Open sea	−0.0701	0.0494	0.0687
cg24130711	2	*CCDC85A*	gene body	Shore	−0.0541	0.0494	0.0596
cg26175971	2	*CYP27A1*	gene body	Island	−0.052	0.0494	0.1145
cg26874367	2		intergenic	Open sea	−0.0904	0.0494	**0.0220**
cg01615258	3		intergenic	Shore	−0.0521	0.0494	**0.0202**
cg01814969	3	*QARS*	gene body	Shore	−0.0468	0.0498	0.9991
cg15450445	3		intergenic	Open sea	−0.0141	0.0143	0.9991
cg24840062	3	*CDCP1*	gene body	Open sea	−0.1073	0.0494	**0.0218**
cg25616514	3		intergenic	Open sea	−0.0315	0.0498	**0.0399**
cg03002688	4		intergenic	Open sea	−0.0598	0.0498	**0.0137**
cg18860310	4	*SLC10A6*	gene body	Open sea	−0.05	0.0494	0.0946
cg26994377	4		intergenic	Open sea	−0.0587	0.0494	0.4133
cg00608540	5	*TRIM7*	gene body	Open sea	−0.0388	0.0498	**0.0319**
cg01204911	5	*ARHGAP26*	gene body	Open sea	−0.0406	0.0494	0.0949
cg06527989	5	*UNC5A*	gene body	Shore	−0.0735	0.0494	**0.0291**
cg07475151	5	*LOC100268168*	gene body	Open sea	−0.1005	0.0494	0.0648
cg11953913	5	*C5orf32*	5'UTR	Open sea	−0.0542	0.0494	0.6035
cg21550107	5		intergenic	Open sea	−0.0261	0.0494	0.9987
cg02174359	6	*MRPS18A*	gene body	Shore	−0.0494	0.0494	0.9963
cg04334016	6	*CNKSR3*	gene body	Open sea	−0.0324	0.0494	0.3819
cg09639771	6		intergenic	Open sea	−0.0442	0.0494	0.9975
cg02377685	7	*GBX1*	TSS200	Island	−0.0645	0.0494	0.9980
cg03761471	7	*ZYX*	TSS1500	Shore	−0.0661	0.0494	0.1001
cg04153882	7	*WIPI2*	gene body		0.0659	0.0494	0.1738
cg15602580	7	*SDK1*	gene body	Shore	−0.0553	0.0494	**0.0258**
cg18967180	7	*DENND2A*	gene body		−0.068	0.0494	0.9978
cg21252523	7		intergenic	Shore	−0.0394	0.0494	**0.0345**
cg27403098	7	*KIAA1908*	gene body		−0.0119	0.0498	0.1343
cg14681767	8	*ARHGEF10*	5'UTR	Island	−0.0913	0.0494	0.0718
cg17588094	8		intergenic	Shelf	−0.0167	0.0498	0.9987
cg05616472	9	*EHMT1*	gene body		−0.0475	0.0494	0.9983
cg14223444	9	*TSTD2*	5'UTR	Shore	−0.0367	0.0498	**0.0253**
cg01311537	10	*C10orf128*	gene body	Open sea	−0.026	0.0494	**0.0350**
cg08560387	10	*TSPAN14*	5'UTR		−0.1142	0.0498	**0.0174**
cg14770293	10		intergenic	Island	−0.0763	0.0494	0.9992
cg24168991	10	*ITPRIP*	5'UTR	Shelf	−0.0308	0.0494	**0.0198**
cg03611487	11	*LOC100126784*	gene body	Shelf	−0.0486	0.0494	**0.0149**
cg10141801	11	*GUCY2E*	TSS200	Open sea	−0.0456	0.0494	0.9599
cg13100962	11		intergenic	Open sea	−0.0544	0.0498	**0.0233**
cg17679104	11	*STK33*	TSS1500	Island	−0.0612	0.0494	**0.0351**
cg22623080	11	*AMOTL1*	TSS200	Island	−0.0437	0.0498	**0.0314**
cg23460961	11		intergenic	Open sea	−0.0752	0.0494	**0.0227**
cg04890607	12	*HMGA2*	gene body	Open sea	−0.1331	0.0498	**0.0356**
cg27292547	12		intergenic	Open sea	−0.0539	0.0498	**0.0339**
cg11398020	13	*KLF5*	gene body	Island	−0.0766	0.0494	0.0667
cg01972418	14	*PAX9*	TSS1500	Island	−0.0386	0.0498	**0.0272**
cg18581173	15	*CT62*	TSS1500	Shore	−0.0673	0.0494	0.9997
cg20172862	15		intergenic	Island	−0.0309	0.0498	0.9981
cg24359188	15	*BUB1B*	TSS200		−0.0758	0.0494	0.1524
cg26568226	15	*CYFIP1*	5'UTR	Island	0.0733	0.0494	**0.0470**
cg04470044	16	*WFDC1*	gene body	Shelf	−0.0259	0.0498	**0.0274**
cg10155261	16	*LOC23117*	gene body	Open sea	−0.0499	0.0494	0.0581
cg26591162	16	*SRL*	3'UTR	Open sea	−0.0149	0.0494	0.9956
cg07777703	17	*TUBG2*	gene body	Shore	−0.0491	0.0498	0.9984
cg07848706	17		intergenic	Island	−0.0722	0.0494	0.9987
cg08960549	17		intergenic	Open sea	−0.0198	0.0494	0.9984
cg09580608	17	*GNA13*	1stExon	Island	−0.0852	0.0494	**0.0142**
cg22163463	17	*PITPNM3*	gene body	Shore	−0.056	0.0494	**0.0489**
cg01823541	19	*GNG7*	5'UTR	Island	−0.0744	0.0498	0.0641
cg03363633	19	*TYROBP*	TSS1500	Open sea	−0.0515	0.0494	**0.0185**
cg22313519	19	*KIAA1683*	TSS1500		−0.0386	0.0498	**0.0496**
cg20477147	20	*NPEPL1*	gene body	Shore	−0.0712	0.0494	**0.0211**
cg26468205	20	*PCMTD2*	TSS200	Island	−0.015	0.0494	**0.0487**

Bold values indicate adjusted *P*‐value < 0.05.

* *P* value adjusted by age, genetic ancestry, smoking status, and cellular heterogeneity.

It has been shown that age, genetic ancestry, smoking status, and possible disease‐related cell heterogeneity in blood may act as potential cofounders when investigating DNA methylation differences between cases and controls. Adjusting our analysis for these epidemiological variables and the predicted cell‐type proportion in leukocytes, 38 of the CpGDMs still showed significant differences between BCP and healthy women (*P* < 0.05 adjusted, Table 1). This panel of 38 candidate CpGDMs sites also groups samples by separating cancer patients from healthy women, using cluster analysis (Fig. 1B).

Most of the candidate CpGDMs (37 of 38) were less methylated in the leukocyte DNA from cancer patients compared to controls, with only one CpGDMs hypermethylated. Out of 38 CpGDMs, 29 were associated with 28 different genes: 11 in gene promoters and 18 in gene bodies (Fig. 1C). The remaining nine CpGDMs mapped to intergenic regions. Considering density and regional composition of CpG, the majority (81.6%) of CpGDMs was located outside CpG‐rich regions (CpG islands), with 34.2% located in CpG shores flanking the islands (Fig. 1C). The genomic distribution of the CpGDMs in relation to gene context (promoter, UTR, 1st exon, body gene, or intergenic region) was not different compared to the whole array CpG distribution (Fisher exact test, *P* = 0.480) (Table S5). Gene ontology analysis revealed a functional enrichment of candidate CpGDMs in biological processes associated with signal transduction (GO:0007165) (Fisher exact test, *P* = 0.024) and regulation of stem cell population maintenance (GO:2000036) (Fisher exact test, *P* < 0.01).

Among the candidate CpGDMs, we found eight genes with differential methylation previously associated with breast cancer susceptibility and pathology in the G2SBC and COSMIC Databases (*AMOTL1*, *CDCP1*, *CYFIP1*, *MAP3K6*, *MIB2*, *SDK1*, *TAL1*, and *TYROBP*) (Table S6). Broadening the search to genes previously associated with other cancer types in *Cancer* *census*, we identified three other genes overlapping with CpG sites identified as candidate CpGDMs (*HMGA2*, *GNA13*, and *STK33*) (Table S6). Except *CYFIP1*, all CpGDMs in identified cancer genes are in average hypomethylated in leukocytes of BCP compared to healthy women. Furthermore, five CpGDMs that show an average methylation difference > 10% between patients and controls mapped to four different genes: *CDCP1*, *MIB2*, *TSPAN14*, and *HMGA2*, three of which were previously described as cancer genes (Table 1).

To validate methylation status determined by the microarray, we analyzed in four patients and four control methylation status of four candidate CpGDMs and their flanking regions by bisulfite‐treated DNA sequencing. Selection criteria were CpGDMs located in regulatory regions of genes previously associated with breast cancer. Hypermethylation of *CYFIP1* gene (only CpGDM hypermethylated in patients) was observed in patients with breast cancer (*P* = 2.22 × 10^−12^), while *MAP3K6* and *MIB2* gene promoters showed a slight hypomethylation in BCP (Fig. S2).

Next, we studied whether methylation at leukocytes could recapitulate concomitant methylation changes in the primary tumor. Because methylation data on primary breast tissues were not available for the Uruguayan BCP and there are no public methylation profiling data of Latin American patients, an independent public cohort of European BCP was used to validate *in* *silico* the CpG differential methylation previously identified in blood (Table S3). We analyzed methylation data of the 38 CpGDMs panel in primary breast tumors samples and their paired‐normal breast tissue of this independent cohort. We found that the 38 CpGDMs previously identified in blood were capable of clustering tumor breast samples and normal tissues separately using hierarchical cluster analysis (Fig. 1D). More interestingly, methylation levels of 38 CpGDMs were able to separate primary tumor tissue from normal breast tissue in patients with familial breast cancer mutation in the *BRCA2* gene (pairs N4/T4, N5/T5, N11/T11) (Fig. 1D, Table S3). In addition, 9 of the 38 candidate CpGDMs showed differential methylation between tumor tissues and paired healthy tissues in the European cohort (Wilcoxon test, FDR adjusted *P*‐value < 0.05; Table S7).

### 3.2 Validation of aberrant CpG methylation at the *CYFIP1* gene as a sporadic breast cancer candidate biomarker

**Fig. 2:**
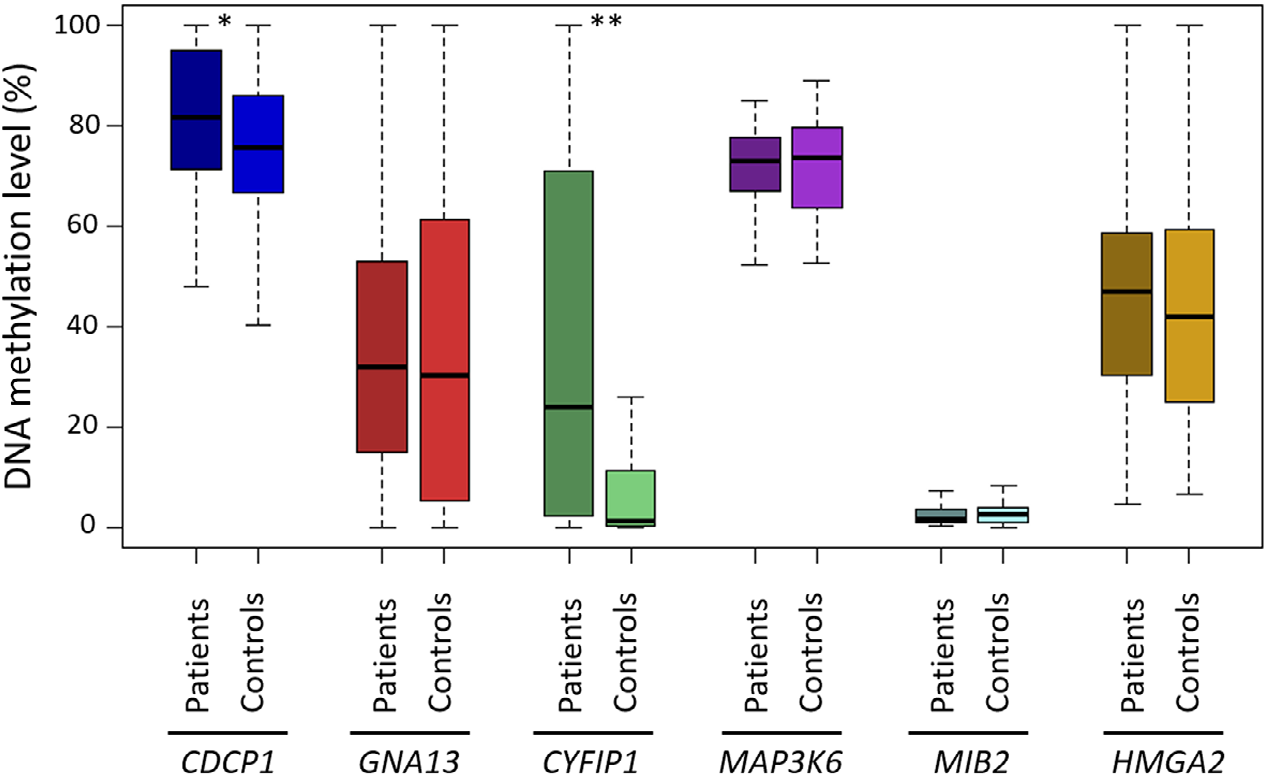
Validation of methylation differences at candidate genes in an independent cohort (validation cohort). DNA methylation level of *MAP3K6*, *CYFIP1*, *HMGA2*, *GNA13*, *MIB2*, and *CDCP1* genes in leukocytes from 80 BCP and 80 healthy controls. The boxes represent the interquartile ranges, and the lines across the boxes indicate the median value. Statistically significant differences between cancer patients and healthy controls were determined using Wilcoxon rank sum test (*P < 0.05; **P < 0.01).

With the aim to simplify the CpGDM panel for a more suitable use in a clinical environment, we selected seven candidate CpGDMs sites located at regulatory sites of genes previously reported as associated with cancer and/or having an average methylation difference > 10% between patients and controls. These CpG sites correspond to the following selected regions: *MAP3K6* promoter region (cg14024502), 5′UTR of *CYFIP‐1* (cg26568226), *HMGA2* body gene (cg04890607), 1st exon of *GNA13* (cg09580608), *MIB2* promoter region (cg01229567, cg19246761), and intron 1 of *CDCP1* (cg24840062). To validate the differentially methylated regions selected, we used a larger and independent Uruguayan leukocyte cohort of 80 sporadic BCP and 80 healthy controls using MS‐HRM PCR assay (Table S2). *CYFIP1* and *CDCP1* were differentially methylated in patients compared to controls in the validation cohort (Wilcoxon test, *P* < 0.01 and *P* < 0.05 respectively, Table 2 and Fig. 2). Methylation analysis of 147 bp containing nine CpG sites in the evaluated region of the 5′UTR of *CYFIP1* showed significant consistent DNA hypermethylation in the cancer patients compared with healthy controls (Fig. 2), while analysis of 124 bp containing two CpG sites in intron 1 of *CDCP1* showed significant hypomethylation in cancer patients. Next, we analyzed whether methylation levels in these regions were associated with some epidemiological variables. Adjusting our analysis for age, smoking status, and genetic ancestry with methylation levels in evaluated regions of the six candidate genes selected, *CYFIP1* still showed a significant difference between BCP and healthy women (*P* = 6.1 × 10^−6^, Table 2).

**Table 2:** DNA methylation in candidate genes evaluated in the independent validation cohort. TSS1500: 1500 bp of the start site of transcription.

Gene	Genomic context	Methylation breast cancer (mean)	Methylation controls (mean)	*P* value	*P* value adjusted*
*MAP3K6*	TSS1500	0.717	0.710	0.915	0.799
*CYFIP1*	5′−UTR	0.39	0.10	**7.29 × 10^−7^**	**6.1 × 10^−6^**
*HMGA2*	Body	0.469	0.433	0.277	0.515
*GNA13*	1st exon	0.0035	0.0035	0.878	0.947
*MIB2*	TSS1500	0.024	0.030	0.100	0.128
*CDCP1*	Body	0.807	0.758	**0.044**	0.088

Bold values indicate adjusted *P*‐value < 0.05.

* *P* value adjusted by age, smoking status, and genetic ancestry.

**Fig. 3:**
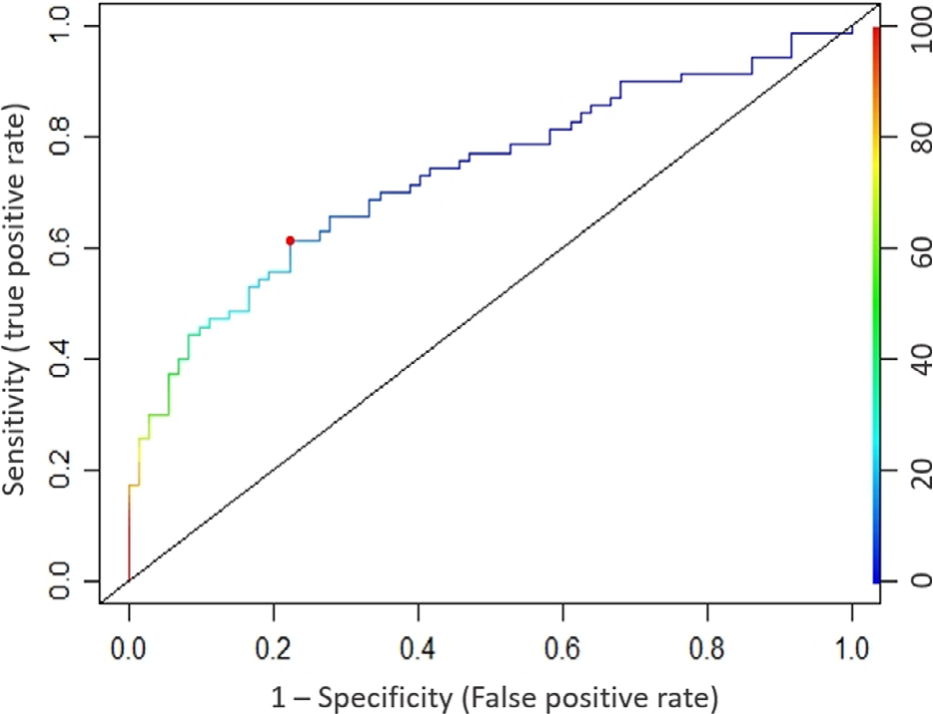
ROC curve assessing the discriminative power of the methylation in 5′UTR region of *CYFIP1* for validation cohort. Area under the curve (AUC) of 0.732 (95% CI: 0.649–0.815), with an optimal cutoff point of 14.28% of methylation (red point).

In order to study *CYFIP1* as a candidate sporadic breast cancer biomarker in blood, we evaluated the strength to predict BCP against controls using ROC curves in the validation cohort. Methylation of candidate CpGDM and the flanking region in the 5′UTR of *CYFIP1* showed a good predictive ability with an area under the ROC curve (AUC) of 0.732 (95% CI, 0.649–0.815) (Fig. 3).

With the aim to elucidate the possible functional role of our candidate biomarker in breast cancer, we analyzed *in silico* methylation data of *CYFIP1* (cg26568226) in tissue samples from BCP from TCGA Program. Comparing 735 primary breast tumors with 89 healthy breast tissues, we detected significant differential methylation of the candidate CpG site located in *CYFIP1* (Wilcoxon test, *P* value = 9.3 × 10^−4^, Table S8, Fig. S3). This result suggested a potential functional role of CYFIP1 methylation in breast cancer development. Next, we wondered whether *CYFIP1* could be contributing to the overall survival of patients with breast cancer. Since we do not have follow‐up data on Uruguayan patients, we studied overall survival in BCP from the TCGA database in relation to *CYFIP1* methylation in primary samples and age at diagnosis of the pathology (Fig. S4). The results of these analysis (Cox Regression, *P* value = 0.681) suggest that 5′UTR *CYFIP1* methylation would not be a prognostic marker for BCP.

## 4 Discussion

Patients with localized breast cancer have a 5‐year survival rate of 98%. However, if it is diagnosed after metastasis, the survival rate drops dramatically to 27%. These results mark the benefit of screening and early detection, and the vital importance of finding new markers to supplement mammography results. For sporadic breast cancer, a variety of changes in DNA methylation were detected both in primary cancer samples [27] and in blood of BCP [11, 13, 28]. In spite of the preclinical data, a meta‐study to find standard candidate markers with potential clinical use has not been performed. These difficulties may be attributable to differences in the subject populations and tumor pathologies, but most likely due to low power in each of these studies. Genetic ancestry should also be considered especially in populations with a high admixed genetic background like Latino ones [4, 29].

In this line, our study described for the first time DNA methylation profiling in leukocytes from sporadic BCP in a Latin American population. Accordingly with the described global hypomethylation in primary breast cancer tissues and in peripheral blood of BCP [4], we have also detected almost exclusively hypomethylated CpGDMs in leukocytes taken from cancer patients. Each tissue has a unique epigenetic signature, which often reflects its differential function [30, 31]. Although the greatest variation in DNA methylation is observed between tissues, interindividual differences in DNA methylation of internal tissues are correlated with blood cells for a group of CpG sites [27, 32, 33, 34]. This reinforces the hypothesis that variation in DNA methylation at a systemic level in many tissues could be associated with predisposition to certain diseases, allowing to detect differential DNA methylation in blood associated with breast cancer several years before diagnosis [35, 36]. Furthermore, in studies like our work that use breast cancer cases recruited at diagnosis we must consider the possibility that the cancer itself is causing the epigenetic changes detected in the blood DNA, including circulating tumor DNA. Unfortunately, primary tumor samples from the Uruguayan BCP were not available. As a closer approach, we confirmed that the panel of 38 CpGDMs detected in leukocytes could be validated in breast cancer tissue of women of European origin, which supports the sensitivity of our current approach using non‐invasive specimens.

To our knowledge, DNA methylation blood biomarkers associated with breast cancer have not been previously described in Latin American countries. If we compared the panel of 38 CpGDMs detected in the Uruguayan population with CpGDMs detected in blood samples of European BCP (GSE37965 [37], GSE51057 [36]), none of our candidate CpGDMs coincides with these panels (data not shown). The different genomic context in admixed populations such as Uruguay as well as different lifestyles could determine differences in epigenetic cancer biomarkers detected. This reinforces the need for each population to detect their own markers associated with breast cancer.

Although most CpGDMs detected have average differences between patients and controls < 10%, these differences are consistent. Blood biomarker identification has the challenge of blood cell‐specific events that cannot be entirely excluded, and the marginal methylation levels introduced by circulating tumor DNA. Therefore, alterations are expected to present changes of small magnitude between cancer patients and controls. Even in the study of Heyn *et al*. [37], in which genetic noise is removed and other sources of confounders are reduced by analyzing identical twin pairs discordant for breast cancer, they detected 403 differentially methylated sites in blood DNA between discordant twins, all with < 8% differences between the two groups. Despite the small change in magnitude, the integration of multiple epigenetic biomarkers as a predictive signature and/or in combination with genetic markers could be of high translational value [9].

Importantly, among the CpGDMs panel detected in leukocytes, CpG sites in genes previously associated with breast cancer are described, including *Cytoplasmic FMR1 interacting protein 1* (*CYFIP1)*, reinforcing the utility of this approach in the search for biomarkers associated with breast cancer in peripheral blood. *CYFIP1* may play an important role in the occurrence and development of cancers. Loss of *CYFIP1* expression has been found in a number of human cancers, including breast cancer, colon cancer, lung cancer, bladder cancer, cutaneous squamous cell carcinoma, nasopharyngeal carcinoma, and acute lymphoblastic leukemia [38, 39, 40, 41]. *CYFIP1* expression was correlated with tumor progression in epithelial cancers and it raised the possibility that loss of CYFIP1 might correlate with clinical outcome [39]. Specifically, CYFIP1 would play a role in the suppression of breast cancer cell migration/invasion and metastasis [42], although its suppressive role has been contradicted in other studies [43]. In sum, the functional role of CYFIP1 in tumor development is still unclear and controversial.

Finally, we are aware that our study should be complemented with validation of the proposed candidate on large sample size. Although methylation of candidate CpGDM and flanking region in 5′UTR of *CYFIP1* in blood showed a good predictive ability of sporadic BCP against healthy women, additional epidemiological factor information including genetic factors and age and is needed to evaluate its potential value as an independent biomarker. The cost of the methylation array‐based technologies limits its use as a screening tool, but the identification of a panel of a limited number of CpG sites as a cancer biomarker would allow evaluation of it with other less expensive technologies.

## 5 Conclusions

In summary, this work represents the first study in Latin America that describes the search for epigenetic markers in peripheral blood in a well‐characterized cohort of patients with sporadic breast cancer. Although not yet adequate for use in clinical settings, the description of a panel of 38 CpGDM associated with breast cancer in the discovery sample and the validation of *CYFIP1* as candidate biomarker in a larger sample demonstrates the potential of blood DNA methylation for development of non‐invasive applications for detection of sporadic breast cancer biomarker in a Latin American population. Future studies should be aimed at continued exploration of blood DNA methylation biomarkers using prospective studies.

## Conflict of interest

The authors declare no conflict of interest.

## Author contributions

MC and MB conceived and designed the project. MC, LF, and ML performed epigenetic experiments, and analyzed and interpreted the experimental data. LB and CB contributed in the selection of samples and in epidemiological data analysis. NA provided the human samples and clinical expertise. ME provided feedback on the epigenetic analyses and the manuscript and provided data. BB contributed in the statistical analysis and provided feedback on manuscript; and MC and MB wrote the manuscript. All authors read and approved the manuscript.

### Peer Review

The peer review history for this article is available at https://publons.com/publon/10.1002/1878‐0261.12842.

## Supporting information


**Fig. S1.** Unsupervised analysis of CpG methylation in breast cancer and healthy controls.
**Fig. S2.** Bisulfite sequencing of *CYFIP1*, *MAP3K6* and *MIB2* genes in sporadic breast cancer and controls.
**Fig. S3.** DNA methylation level of candidate CpGDM site in *CYFIP1* gene (cg26568226) in 735 primary breast tumours and 89 healthy breast tissues from The Cancer Genome Atlas Program (TCGA).
**Fig. S4.** Overall survival analysis of 735 breast cancer patients from The Cancer Genome Atlas (TCGA) database in relation to the methylation in our candidate CpGDM in *CYFIP1*.

**Table S1.** Clinical and epidemiological characteristics of sporadic breast cancer patients (SBC) and healthy controls (C) analyzed on 450K Human Methylation BeadChip platform (discovery cohort).
**Table S2.** Clinical and epidemiological characteristics of sporadic breast cancer patients and healthy controls used as an independent sample to validate candidate CpG sites (validation cohort).
**Table S3.** Clinical characteristics of tumors corresponding to paired breast tissues (Normal/Tumor) of an independent European cohort of breast cancer patients.
**Table S4.** Primers used in bisulfite DNA sequencing and MS‐HRM for the validation of methylation status in selected CpGDMs.
**Table S5.** Comparison of the genomic distribution of 38 CpGDMs to the expected distribution according to all printed CpG in the 450K HumanMethylation array.
**Table S6.** CpGDMs overlapping genes previously associated with cancer (Cancer Gene Census and G2SBC Database).
**Table S7.** Overlapping CpGDMs detected in the blood of breast cancer patients from Latino population and in primary tissues from an independent European cohort of breast cancer patients.
**Table S8.** *In silico* methylation analysis of selected candidate CpGDMs in breast primary tumors (*n* = 735) and normal breast tissues (*n* = 89) of TCGA database.

## Data Availability

The DNA methylation microarray data reported in this article can be found at Gene Expression Ommibus (GEO) under the accession number GSE148663.
