# Supplementary material for: Discovery of novel DNA methylation biomarkers for non‐invasive sporadic breast cancer detection in the Latino population
Source: Mol Oncol. 2020 Nov 19;15(2):473–86. doi: 10.1002/1878-0261.12842 (PMC7858097; doi:10.1002/1878-0261.12842)
Supplement: Supplementary file 1 — Fig. S1. Unsupervised analysis of CpG methylation in breast cancer and healthy controls. Fig. S2. Bisulfite sequencing of CYFIP1, MAP3K6 and MIB2 genes in sporadic breast cancer and controls. Fig. S3. DNA methylation level of candidate CpGDM site in CYFIP1 gene (cg26568226) in 735 primary breast tumours and 89 healthy breast tissues from The Cancer Genome Atlas Program (TCGA). Fig. S4. Overall survival analysis of 735 breast cancer patients from The Cancer Genome Atlas (TCGA) database in relation to the methylation in our candidate CpGDM in CYFIP1. [file MOL2-15-473-s001.pdf]

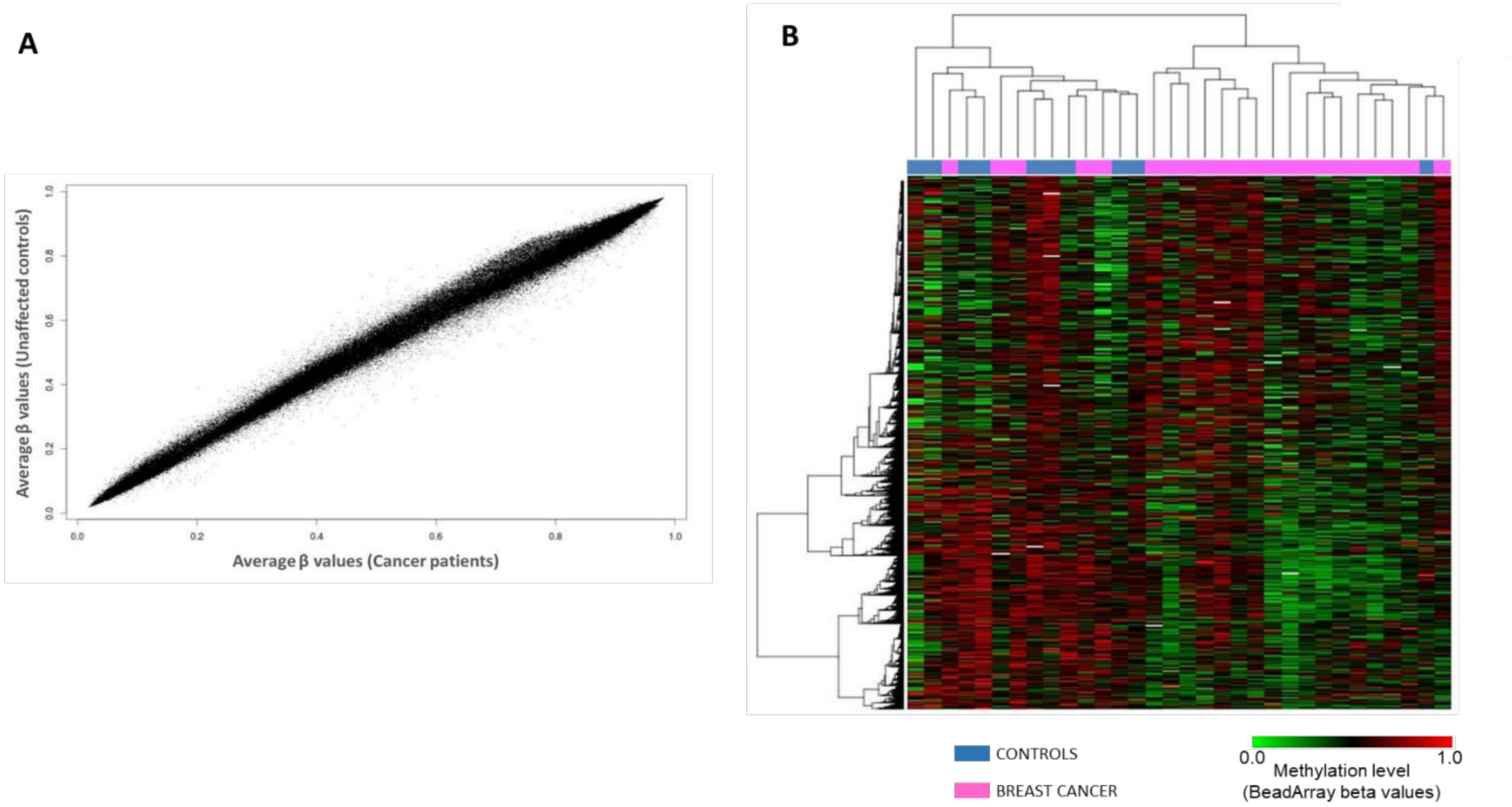

Fig. S1. Unsupervised anlaysis of CpG methylation in breast cancer and healthy controls. **A.** DNA methylation level of CpG sites identified by the Infinium 450K DNA methylation assay. Displayed are averages of normalized beta-values of each CpG in 22 breast cancer patients (x-axis) and 10 unaffected controls (y-axis). **B.** Hierarchical heatmap clustering of random 45,000 CpG sites in 22 breast cancer patients (purple) and 10 healthy controls (blue) analyzed on the Infinium HumanMethylation450 BeadChip platform. We used «complete» method and euclidean distances. Methylation level is color coded (Green: lowest methylation level; red: highest methylation level).

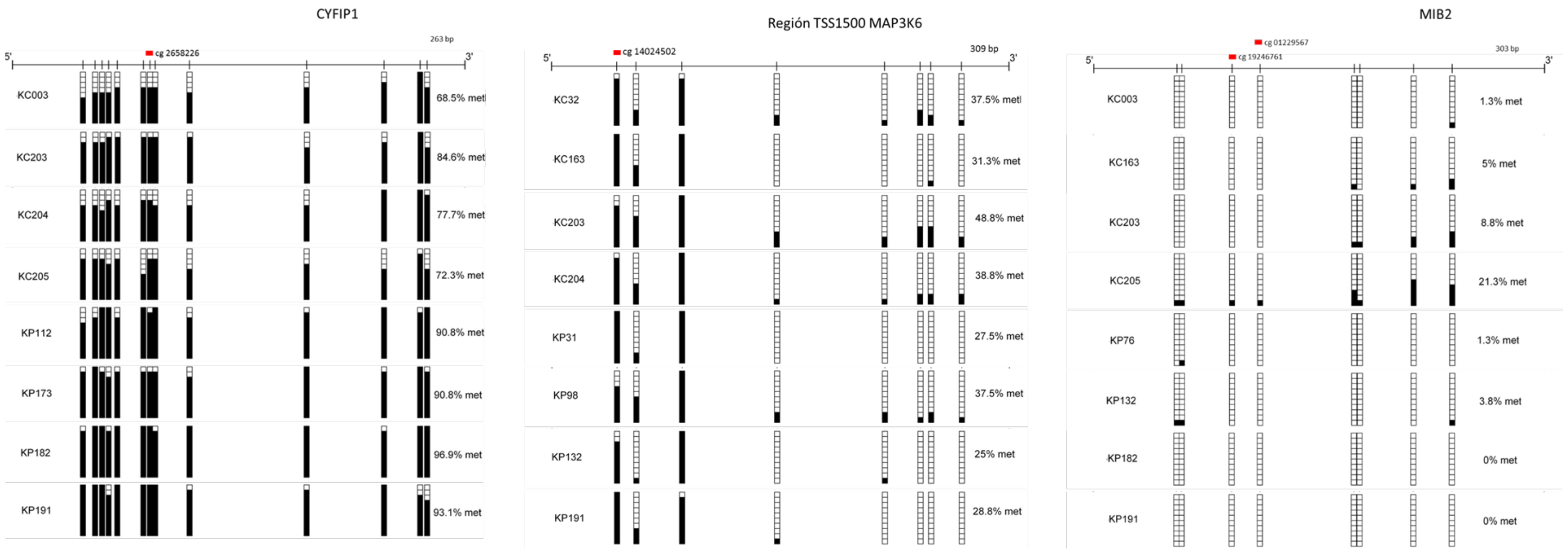

Fig. S2. Analyzed region and results of the bisulfite genomic sequencing of flanking regions of CpG site evaluated by cg2658226 probe in *CYFIP1* gene, cg14024502 probe in *MAP3K6* gene and by cg19246761 and cg01229567 probes in *MIB2* gene. Each row of squares represents a single cloned allele, and each square represents a single CpG site (open square, non-methylated cytosines; filled square, methylated cytosines). The average percentage of methylation of the region evaluated for each individual was indicated.

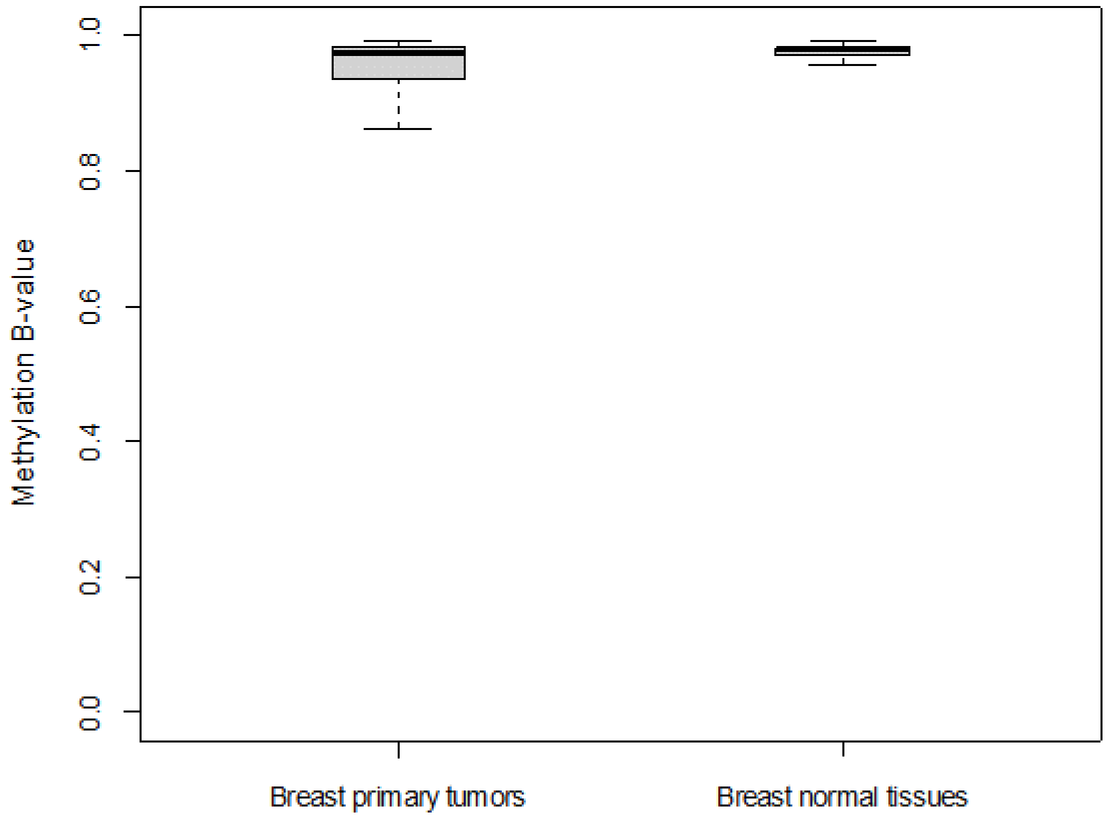

Fig. S3. DNA methylation level of candidate CpGDM site in *CYFIP1* gene (cg26568226) in 745 primary breast tumours and 89 healthy breast tissues from The Cancer Genome Atlas Program (TCGA). The boxes represent the interquartile ranges and the lines across the boxes indicates the median value. Statistically significant differences between primary breast tumours and normal breast tissues were determined using Wilcoxon Rank Sum test ($p<0.01$).

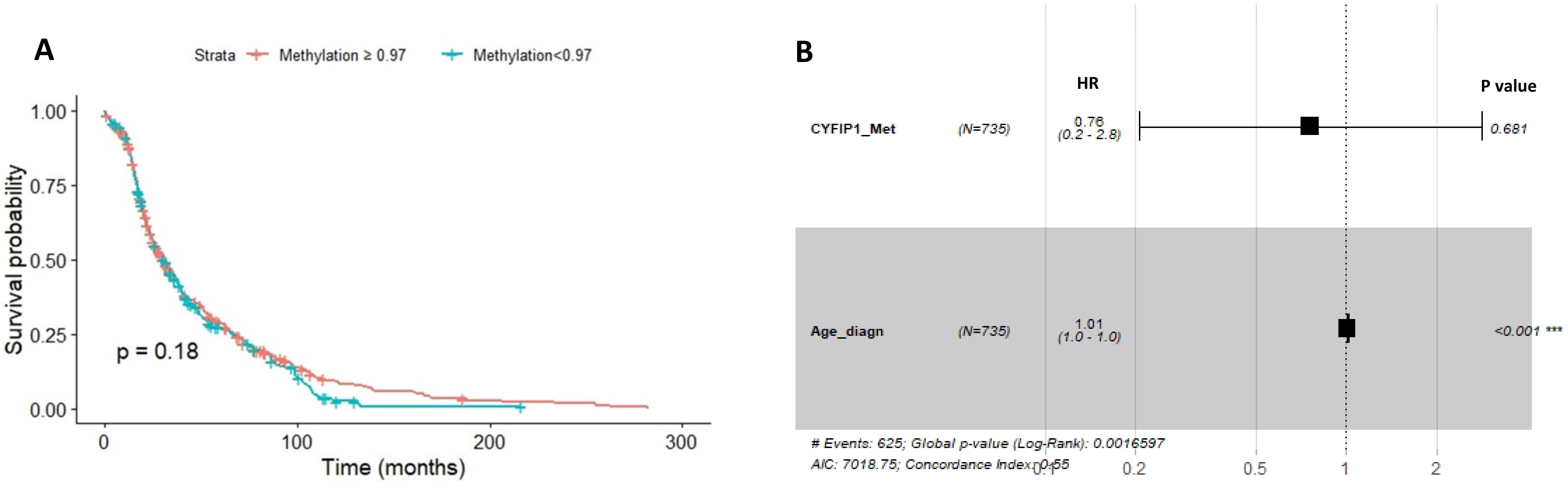

Fig. S4. Overall survival analysis of 735 breast cancer patients from The Cancer Genome Atlas (TCGA) database in relation to the methylation in our candidate CpGDM in *CYFIP1*. **A.** Kaplan Meier analysis grouping patients with methylation values above and below the median (0.97). **B.** Cox Proportional Hazards Models considering methylation in CYFIP1 and age at diagnosis of the pathology. CIFIP1_Met: methylation of cg26568226 in *CYFIP1* gene; Age_diagn: Age at diagnosis of breast cancer; HR: Hazard ratio.
