# Supplementary material for: Discovery of novel DNA methylation biomarkers for non‐invasive sporadic breast cancer detection in the Latino population
Source: Mol Oncol. 2020 Nov 19;15(2):473–86. doi: 10.1002/1878-0261.12842 (PMC7858097; doi:10.1002/1878-0261.12842)
Supplement: Supplementary file 2 — Table S1. Clinical and epidemiological characteristics of sporadic breast cancer patients (SBC) and healthy controls (C) analyzed on 450K Human Methylation BeadChip platform (discovery cohort). Table S2. Clinical and epidemiological characteristics of sporadic breast cancer patients and healthy controls used as an independent sample to validate candidate CpG sites (validation cohort). Table S3. Clinical characteristics of tumors corresponding to paired breast tissues (Normal/Tumor) of an independent European cohort of breast cancer patients. Table S4. Primers used in bisulfite DNA sequencing and MS‐HRM for the validation of methylation status in selected CpGDMs. Table S5. Comparison of the genomic distribution of 38 CpGDMs to the expected distribution according to all printed CpG in the 450K HumanMethylation array. Table S6. CpGDMs overlapping genes previously associated with cancer (Cancer Gene Census and G2SBC Database). Table S7. Overlapping CpGDMs detected in the blood of breast cancer patients from Latino population and in primary tissues from an independent European cohort of breast cancer patients. Table S8. In silico methylation analysis of selected candidate CpGDMs in breast primary tumors (n = 735) and normal breast tissues (n = 89) of TCGA database. [file MOL2-15-473-s002.pdf]

**Table S1.** Clinical and epidemiological characteristics of sporadic breast cancer patients (SBC) and healthy controls (C) analized on 450K HumanMethylation BeadChip platform (discovery cohort). BMI: body mass index, N/A: not applicable.

| Variable | Breast cancer patients | Healty controls | P value |
|---|---|---|---|
| **Age at diagnosis/recruitment (years)** | | | |
| mean ± SD | 59 ± 9.2 | 62.6 ± 7.9 | 0.293 |
| **BMI (kg/m2)** | | | |
| mean ± SD | 29.4 ± 4.9 | 28.6 ± 5.6 | 0.716 |
| **Genetic ancestry (%) mean ± SD** | | | |
| European | 76.4 ± 11.5 | 79.0 ± 12.8 | 0.580 |
| African | 9.4 ± 7.7 | 6.6 ± 6.9 | 0.351 |
| Native american | 14.2 ± 11.1 | 14.4 ± 9.7 | 0.967 |
| **Smoking (%)** | | | |
| Yes | 31.8 | 0 | 1 |
| No | 68.2 | 100 | |
| **Tumor stage (%)** | | | |
| I | 26.3 | N/A | |
| II | 57.9 | N/A | |
| III | 15.8 | N/A | |
| IV | 0 | N/A | |
| **Histological type (%)** | | | |
| Ductal | 84.2 | N/A | |
| Lobular | 10.5 | N/A | |
| Papilary | 5.3 | N/A | |
| **Estrogen receptor (%)** | | | |
| Positive | 77.8 | N/A | |
| Negative | 22.2 | N/A | |
| **Progesterone receptor (%)** | | | |
| Positive | 66.7 | N/A | |
| Negative | 33.3 | N/A | |
| **Her2 (%)** | | | |
| Positive | 38.9 | N/A | |
| Negative | 61.1 | N/A | |

**Table S2.** Clinical and epidemiological characteristics of sporadic breast cancer patients and healthy controls used as an independent sample to validate candidate CpG sites (validation cohort). N/A: not applicable.

| Variable | Breast cancer patients | Healty controls | P value |
|---|---|---|---|
| **Age at diagnosis/recruitment (years)** | | | |
| mean ± SD | 55.9 ± 10.0 | 53.6 ± 9.7 | 0.147 |
| **BMI (kg/m2)** | | | |
| mean ± SD | 27.7 ± 5.7 | 26.5 ± 5.4 | 0.176 |
| **Genetic ancestry (%) mean ± SD** | | | |
| European | 78.4 ± 12.5 | 76.0 ± 14.1 | 0.258 |
| African | 9.3 ± 7.5 | 9.4 ± 7.7 | 0.955 |
| Native american | 12.3 ± 9.3 | 14.6 ± 10.9 | 0.150 |
| **Smoking (%)** | | | |
| Yes | 53.4 | 51.3 | 0.815 |
| No | 46.6 | 48.7 | |
| **Tumor stage (%)** | | | |
| I | 34.7 | N/A | |
| II | 34.6 | N/A | |
| III | 19.2 | N/A | |
| IV | 11.5 | N/A | |
| **Histological type (%)** | | | |
| Ductal | 84.0 | N/A | |
| Lobular | 14.0 | N/A | |
| Papilary | 2.0 | N/A | |
| **Estrogen receptor (%)** | | | |
| Positive | 66.0 | N/A | |
| Negative | 34.0 | N/A | |
| **Progesterone receptor (%)** | | | |
| Positive | 66.7 | N/A | |
| Negative | 33.3 | N/A | |
| **Her2 (%)** | | | |
| Positive | 28.9 | N/A | |
| Negative | 71.1 | N/A | |

**Table S3.** Clinical characteristic of tumors corresponding to paired breast tissues (Normal/Tumor) of independent European cohort of breast cancer patients. TNP: triple negative phenotype, N: normal breast tissue, T: breast tumor tissue, NA: data not available.

| Paired tissues | Subtype | Grade | Tumor stage | Familial |
|---|---|---|---|---|
| N1/T1 | Luminal A | 1 | IIa | No |
| N2/T2 | Luminal A | 1 | I | No |
| N3/T3 | Basal | 2 | IIa | No |
| N4/T4 | Luminal B | 3 | IIIa | BRCA2 mut |
| N5/T5 | TNP | 3 | IIa | BRCA2 mut |
| N6/T6 | Basal | 3 | NA | No |
| N7/T7 | Basal | NA | NA | No |
| N8/T8 | Basal | NA | I | No |
| N9/T9 | Basal | 3 | I | No |
| N10/T10 | Luminal A | NA | IIb | No |
| N11/T11 | Basal | 2 | IIa | BRCA2 mut |
| N12/T12 | Basal | 3 | IIIb | No |

**Table S4.** Primers used in bisulfite DNA sequencing and MS-HRM for the validation of methylation status in selected CpGDMs.

| Probe | Gene | Region | N° evaluated CpGs | Primer | Sequence |
|---|---|---|---|---|---|
| | **Bisulfite sequencing** | | | | |
| cg26568226 | *CYFIP1* | 5´-UTR | 13 | BS_CYFIP1-F | 5' TTGAGAGGAGAATTTGAGAG 3' |
| | | | | BS_CYFIP1-R | 5' ACTAAACACCAAACATAACCTC 3' |
| cg14024502 | *MAP3K6* | promoter | 8 | BS_MAP3K6-F | 5' GTTTAGGGTGTAGGTTTTTTTT 3' |
| | | | | BS_MAP3K6-R | 5' TAAAACTCAACCTCTCCCC 3' |
| cg01229567<br>cg19246761 | *MIB2* | promoter | 8 | BS_MIB2-F | 5' TTGTAGGGAAATTTTTTAGGATT 3' |
| | | | | BS_MIB2-R | 5' TATCAACCTAAAAAAAAACCCA 3' |
| | **MS-HRM** | | | | |
| cg26568226 | *CYFIP1* | 5´-UTR | 9 | CYFIP1-F | 5' GGAGAATTTGAGAGGTTGGT 3' |
| | | | | CYFIP1-R | 5' CTAACTACCACCCACCTTATCC 3' |
| cg14024502 | *MAP3K6* | promoter | 4 | MAP3K6-F | 5' TTAGGGTTAGGGAAAAGGTT 3' |
| | | | | MAP3K6-R | 5´CCATCCCAATCCCTCCCTACCTA 3´ |
| cg19246761<br>cg01229567 | *MIB2* | promoter | 4 | MIB2-F | 5´ AGTATTTTAGATAAGTAGTTT 3’ |
| | | | | MIB2-R | 5´ CAAAAACCACTAACTATAAA 3´ |
| cg09580608 | *GNA13* | 1st exon | 10 | GNA13-F | 5' TTGATTAYGTTGTTGTAGATGG 3' |
| | | | | GNA13-R | 5' AAATCCACCTTCCTAAAACAA 3' |
| cg04890607 | *HMGA2* | body gene | 4 | HMGA2-F | 5' TTTTTAAGTAGTAGGTGGTTT 3' |
| | | | | HMGA2-R | 5' CCGAAAAATAATCTAACTCC 3' |
| cg24840062 | *CDCP1* | body gene | 2 | CDCP1-F | 5' GGAATTTTGTTTTATTGTTTTT 3' |
| | | | | CDCP1-R | 5´CTAACTCTTAAACCACTTTAA 3´ |

**Table S5.** Comparison of the genomic distribution of 38 CpGDMs to the expected distribution according to all printed CpG in the 450K HumanMethylation array. TSS1500: 1500 bp of transcription initiation site, TSS200: 200 bp of transcription initiation site, UTR: untranslated region.

| ***Genomic region*** | ***% CpG on microarray*** | ***CpGDM (n)*** | ***Expected CpG (n)*** |
|---|---|---|---|
| TSS1500 | 14.97 | 9 | 5.7 |
| TSS200 | 11.13 | 2 | 4.2 |
| 5´UTR | 11.41 | 5 | 4.3 |
| 1st exon | 6.83 | 1 | 2.6 |
| Gene body | 31.61 | 12 | 12 |
| 3´UTR | 3.44 | 0 | 2.6 |
| Intergenic | 20.59 | 8 | 7.8 |

***** Fisher test p value = 0.480

**Table S6.** CpGDMs overlapping genes previously associated to cancer (COSMIC and G2SBC Database).

| ***Gene*** | ***Cancer type*** | ***Methylation direction*** | ***Genomic context*** | ***CpG island context*** |
|---|---|---|---|---|
| *AMOTL1* | breast | hypomethylation | TSS200 | Island |
| *CDCP1* | breast | hypomethylation | gene body | open sea |
| *CYFIP1* | breast | hypermethylation | 5´UTR | island |
| *GNA13* | lymphoma, gastric cancer | hypomethylation | 1st Exon | island |
| *HMGA2* | lipoma, salivary gland adenoma | hypomethylation | gene body | open sea |
| *MAP3K6* | breast, ovary | hypomethylation | TSS1500 | shore |
| *MIB2* | breast | hypomethylation | TSS1500 | shore |
| *SDK1* | breast | hypomethylation | gene body | shore |
| *STK33* | pancreatic tumour | hypomethylation | TSS1500 | island |
| *TAL1* | breast, ALL | hypomethylation | gene body | island |
| *TYROBP* | breast | hypomethylation | TSS1500 | open sea |

ALL: acute lymphoblastic leukemia, AML: acute mieloblastic leukemia, TSS200: 200 bp from transcription start site, TSS1500: 1500 bp from transcription start site.

**Table S7.** Overlapping CpGDMs detected in the blood of breast cancer patients from Latino population and in primary tissues from an independent European cohort of breast cancer patients.

| CpG | CHR | UCSC REFGENE | Genomic context | CpG island context | P value (FDR) |
|---|---|---|---|---|---|
| cg04400047 | 1 | *UBIAD1* | TSS1500 | shore | 0.007 |
| cg06432479 | 1 | *TAL1* | gene body | island | 0.007 |
| cg26874367 | 2 | | intergenic | open sea | 0.021 |
| cg25616514 | 3 | | intergenic | open sea | $9.84 \times 10^{-9}$ |
| cg01615258 | 3 | | intergenic | shore | $1.12 \times 10^{-4}$ |
| cg01311537 | 10 | *C10orf128* | gene body | open sea | $7.82 \times 10^{-4}$ |
| cg13100962 | 11 | | intergenic | open sea | $9.80 \times 10^{-9}$ |
| cg23460961 | 11 | | intergenic | open sea | 0.028 |
| cg22313519 | 19 | *KIAA1683* | TSS1500 | | $7.79 \times 10^{-4}$ |

**Table S8.** *In silico* methylation analysis of selected candidate CpGDMs in breast primary tumors (n=735) and normal breast tissues (n=89) of TCGA database.

| ***Gene*** | ***cg*** | ***Breast primary tumors Mean ± SD*** | ***Breast normal tissues Mean ± SD*** | ***p value**** |
|---|---|---|---|---|
| *HMGA2* | cg04890607 | 0.597 ± 0.249 | 0.713 ± 0.144 | $2.8 \times 10^{-4}$ |
| *GNA13* | cg09580608 | 0.048 ± 0.026 | 0.059 ± 0.042 | 0.049 |
| *MAP3K6* | cg14024502 | 0.531 ± 0.127 | 0.527 ± 0.054 | 0.735 |
| *CYFIP1* | cg26568226 | 0.946 ± 0.060 | 0.973 ± 0.020 | $9.3 \times 10^{-4}$ |
| *CDCP1* | cg24840062 | 0.720 ± 0.150 | 0.599 ± 0.168 | $6.2 \times 10^{-12}$ |

*Wilcoxon Rank sum test
